# Hydrophobia

**Published:** 1867-05

**Authors:** Amos Sawyer

**Affiliations:** Hillsboro, Ill.


### HYDROPHOBIA.

### SUGGESTION AS TO THE TREATMENT OF HYDROPHOBIA.

I would suggest one grand experiment for the cure of hydrophobia; that is, to trephine and subject the brain to pressure, and thus in a degree suspend the functions of the nervous system. Now when we relieve the excessive nervous irritability, there is reason to hope that nature will be able to eliminate the poison, or else its effects cease before the patient is exhausted. It may be said that this is a dangerous remedy, but a dangerous remedy may be required for a dangerous disease.

AMOS SAWYER.

HILLSBORO, ILL., *March 21st, 1867.*

